# The Role of *Lactobacillus plantarum* in Reducing Obesity and Inflammation: A Meta-Analysis

**DOI:** 10.3390/ijms25147608

**Published:** 2024-07-11

**Authors:** Chen-Pi Li, Chin-Chang Chen, Yao Hsiao, Chieh-Hsin Kao, Chin-Chu Chen, Hao-Jan Yang, Ru-Yin Tsai

**Affiliations:** 1 Department of Nursing, Tung’s Taichung MetroHarbor Hospital, Taichung 43503, Taiwan; t8369@ms.sltung.com.tw; 2 Department of Public Healthy, College of Health Care and Management, Chung Shan Medical University, Taichung 40201, Taiwan; hjyang@csmu.edu.tw; 3 Department of Anatomy, School of Medicine, Chung Shan Medical University, Taichung 40201, Taiwan; geoge6211@csmu.edu.tw; 4 Department of Medical Education, Chung Shan Medical University Hospital, Taichung 40201, Taiwan; 5 School of Medicine, Chung Shan Medical University, Taichung 40201, Taiwan; s1001085@gm.csmu.edu.tw (Y.H.); s1001147@gm.csmu.edu.tw (C.-H.K.); 6 Grape King Bio Ltd., Taoyuan City 32542, Taiwan; gkbioeng@grapeking.com.tw; 7 Department of Family and Community Medicine, Chung Shan Medical University Hospital, Taichung 40201, Taiwan

**Keywords:** probiotic, inflammation, body mass index, visceral fat, cholesterol, blood pressure

## Abstract

Recent research has underscored the efficacy of *Lactobacillus plantarum (L. plantarum)* in managing obesity among healthy adults. This meta-analysis reviewed randomized controlled trials (RCTs) from major databases up to May 2024, focusing on the effects of *L. plantarum* on body weight, body mass index (BMI), and metabolic parameters. This study has been registered in PROSPERO (number: CRD 42024531611). The analysis of nine studies revealed significant weight reduction and BMI decreases with *L. plantarum* supplementation compared to a placebo. Notably, using more than two strains together enhanced these effects. Improvements were also observed in abdominal fat and inflammatory markers such as interleukin-6 (IL-6) and high-sensitivity C-reactive protein (hs-CRP). This meta-analysis synthesizes evidence from nine RCTs to test the hypothesis that *L. plantarum* supplementation effectively reduces body weight and BMI in healthy adults compared to a placebo. However, variations in study designs, probiotic strains, and intervention durations call for more robust trials to confirm these benefits.

## 1. Introduction

The escalating global obesity rates across various age groups represent a critical public health challenge, influenced by a complex array of environmental, genetic, neural, and endocrine factors [1,2,3]. Recent research highlights the significant role of gut microbiota in obesity, presenting new possibilities for microbiota-mediated weight management interventions [4]. Notably, shifts in gut microbiota composition, such as changes in the Bacteroidetes/Firmicutes ratio and levels of specific microbial genera, correlate with obesity [5]. *Lactobacillus*, including *Lactobacillus plantarum*, has shown diverse effects on weight, with some strains potentially aiding in weight management while others may contribute to weight gain [6,7].

*L. plantarum,* often included in health-promoting products, has shown promising effects in clinical settings, such as regulating gastrointestinal functions, reducing serum cholesterol, and boosting immunity [8,9]. Among the key health benefits linked to probiotic consumption is body weight regulation [10]. *L. plantarum*, a common component in probiotic formulations, is particularly noted for its potential influence on metabolic health. Initial studies indicate that *L. plantarum* may help manage weight through various mechanisms, including altering gut microbiota [4], engaging the immune system [11,12], and influencing metabolism [13,14]. Nonetheless, the evidence is still patchy and inconclusive, with inconsistent outcomes reported across different studies.

Recognizing the potential of *L. plantarum* as a therapeutic agent for obesity and related metabolic disorders, it is essential to thoroughly synthesize existing evidence. To date, no meta-analysis has specifically focused on the effects of *L. plantarum* on obesity in healthy adults. This represents a substantial knowledge gap regarding the probiotic’s efficacy and the underlying mechanisms of its action. We hypothesized that *L. plantarum* supplementation would lead to significant weight reduction and improvement in metabolic health indicators compared to a placebo in healthy adults. This systematic review and meta-analysis seek to confirm this hypothesis and identify the underlying mechanisms by which *L. plantarum* exerts its effects, thereby filling a crucial gap in current research on probiotics and weight management.

## 2. Results

### 2.1. Study Search and Characteristics of Included Patients

**Figure 1:**
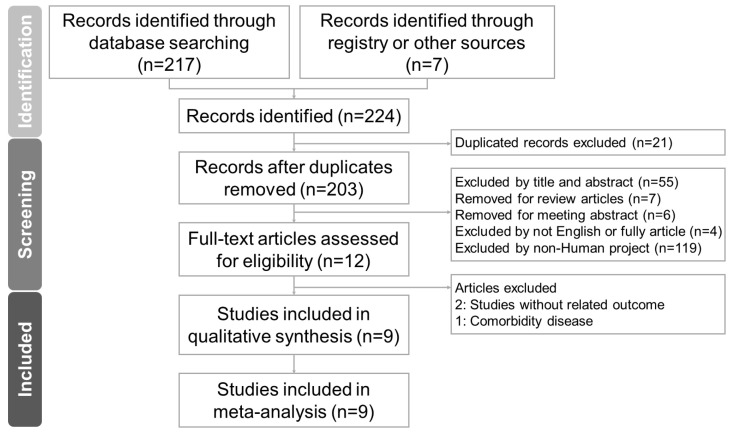
A diagram of the study selection process for the systematic review and meta-analysis on *L. plantarum* reducing body weight and body mass index in healthy adults with obesity. From 224 records, only 9 were eligible and included in the review.

Figure 1 delineates the process of trial screening and selection. We initially explored four databases (PubMed, Scopus, Cochrane Library, and Web of Science) and an additional source, the ‘related articles’ feature in PubMed, which resulted in the identification of 224 trials. Upon the removal of duplicates, a total of 203 trials were subjected to title and abstract screening, leading to the exclusion of 191 trials. A thorough full-text review of the remaining 12 trials led to the further exclusion of 3 trials due to reasons such as unrelated outcomes (2 trials [15,16]) and the presence of comorbid diseases (1 trial [17]). Consequently, nine trials [6,10,11,12,13,18,19,20,21] were incorporated into this meta-analysis. It is noteworthy that all the included studies were published in the English language. Table 1 provides an overview of the fundamental characteristics of the trials under consideration. These nine trials, published in the span of 2012 to 2023, encompassed a total of 667 participants, with the number of participants per trial varying from 44 to 99. In four of these trials, a single-strain variant of *L. plantarum* was utilized [6,11,12,21], while the remaining five trials employed a multi-strain variant of *L. plantarum* [10,13,18,19,20]. The primary focus of all the included trials was to investigate the potential impact of *L. plantarum* on body weight or BMI.

### 2.2. Quality Assessment

**Figure 2:**
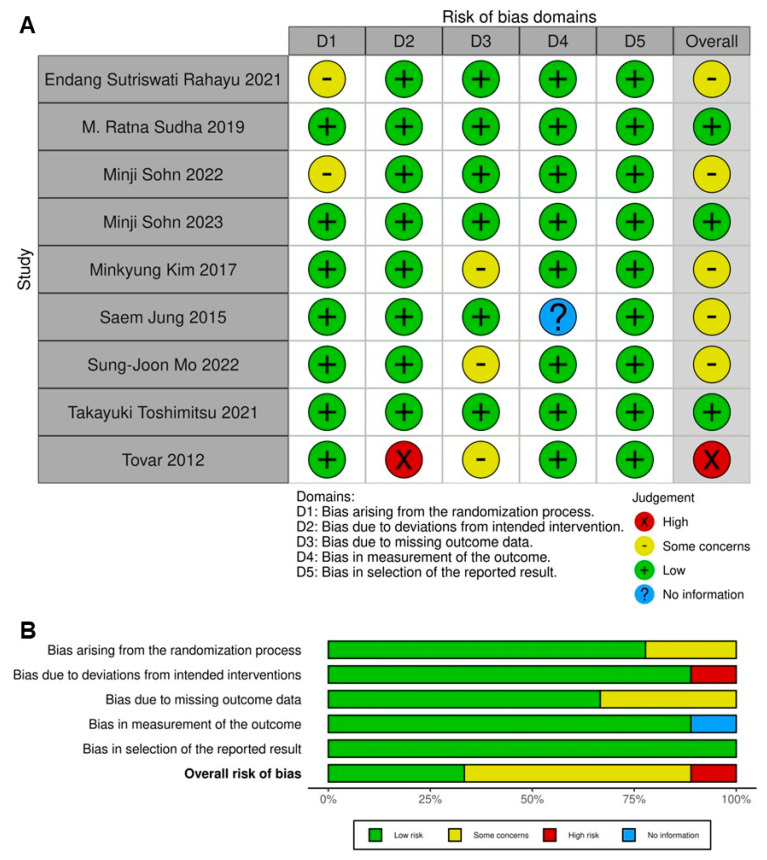
Assessment of methodological quality of the included trials. (**A**) Assessment of risk of bias of each selected article, according to Rob. (**B**) Assessment of overall risk of bias (as percentage) according to intention-to-treat or per protocol studies. Deviations from intended interventions were the main cause of high risk of bias in all the studies, followed by missing outcome data and lack of a randomization process [6,10,11,12,13,18,19,20,21].

Figure 2A,B illustrate the methodological integrity of the trials included in our study. Each trial exhibited a low risk of bias, primarily due to the selection of reported results. In terms of bias stemming from insufficient randomization, seven trials were deemed to have a low risk [10,11,13,18,19,20,21], while two trials raised some concerns due to the absence of detailed information on the randomization process [6,12]. Eight trials were found to have a low risk of bias in relation to deviations from intended interventions [6,12], whereas one trial was identified as having a high risk due to a significant proportion of intervention interruptions [18]. Six trials were assessed to have a low risk of bias due to missing data from dropouts and outcome measurement [6,10,11,12,19,21], while three trials raised some concerns due to a high percentage of missing data and insufficient information on the blinding of the outcome assessor [13,18,20].

### 2.3. Impact of L. plantarum on Body Mass Index and Body Weight

**Figure 3:**
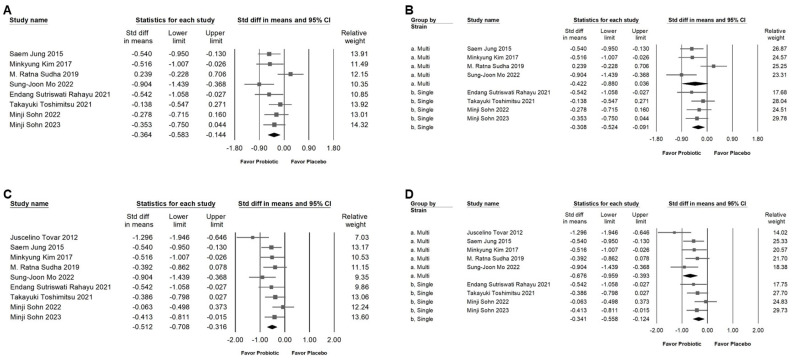
An illustration of the impact of *L. plantarum* consumption, organized into four distinct sections: (**A**) shows the effect on BMI, (**B**) provides a subgroup analysis related to (**A**), (**C**) depicts the influence on body weight, and (**D**) offers a subgroup analysis for (**C**). The weight loss effect induced by *L. plantarum* is indicated by the square (representing the standardized mean difference) moving towards the left. The horizontal line through the square illustrates the 95% confidence interval, while the diamond symbol summarizes the overall effect size [6,10,11,12,13,18,19,20,21].

As shown in Figure 3A,C, patients who were overweight demonstrated that the intervention had a significant effect in downregulated BMI and BW (SMD: −0.364, 95% CI: −0.583 to −0.144; *I*^2^ = 46.795%, *p* = 0.068; SMD: −0.512, 95% CI: −0.708 to −0.316; *I*^2^ = 36.267%, *p* = 0.128, respectively). Interestingly, the subgroup analysis showed a slightly significantly lower BMI (Figure 3B) of the probiotic versus placebo in the *L. plantarum*-alone group (SMD: −0.308, 95% CI: −0.524 to −0.091; *I*^2^ = 0%, *p* = 0.01), but not in the group of the combination with other strains of probiotics (SMD: −0.422, 95% CI: −0.880 to 0.036; *I*^2^ = 73.34%, *p* = 0.676). In terms of BW (Figure 3D), there was a moderate-effect lower BW of the probiotic versus placebo in the multi-strain group (SMD: −0.676, 95% CI: −0.959 to −0.393; *I*^2^ = 37.274%, *p* = 0.173) while there was a small effect in the single-strain group (SMD: −0.341, 95% CI: −0.558 to −0.124; *I*^2^ = 0%, *p* = 0.508). Based on the results, the use of *L. plantarum* in combination with other strains of probiotics appears to have a more significant impact on body weight compared to its effect on BMI.

### 2.4. Impact of L. plantarum on Populations from Different Countries

**Figure 4:**
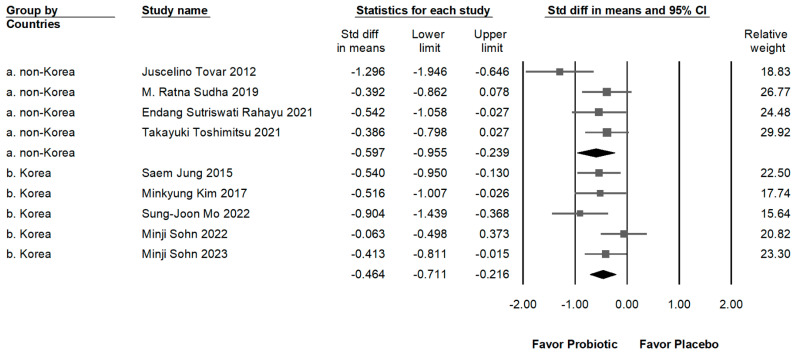
Impact of *L. plantarum* on populations from different countries. The horizontal line through the square illustrates the 95% confidence interval, while the diamond symbol summarizes the overall effect size [6,10,11,12,13,18,19,20,21].

A significant proportion of the trials included in this meta-analysis (55%) were conducted in Korea. It is important to consider that the observed effects of probiotics like *L. plantarum* may vary based on ethno-geographic differences. As shown in Figure 4, the impact of *L. plantarum* does not have a significant difference between non-Korean (SMD: −0.597, 95% CI: −0.950 to 0.239; *I*^2^ = 50.852%, *p* = 0.107) and Korean (SMD: −0.464, 95% CI: −0.711 to 0.216; *I*^2^ = 34.150%, *p* = 0.194) individuals.

### 2.5. Effects of L. plantarum on Blood Lipids, Lipoproteins, and Abdominal Fat Areas

**Figure 5:**
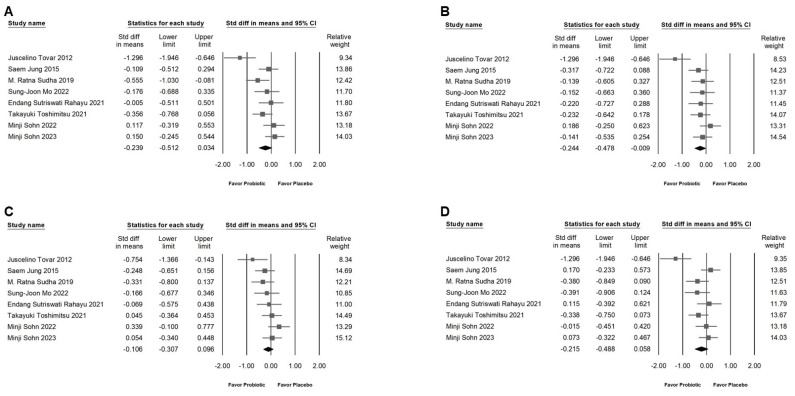
This figure displays a forest plot detailing the effects of *L. plantarum* supplementation on various lipid profiles. This plot is segmented into four distinct sections for clarity: (**A**) illustrates the impact on cholesterol levels, (**B**) explores changes in triglycerides, (**C**) examines alterations in high-density lipoprotein levels, and (**D**) assesses the effect on low-density lipoprotein. The horizontal line through the square illustrates the 95% confidence interval, while the diamond symbol summarizes the overall effect size [6,10,11,12,13,18,19,21].

**Figure 6:**
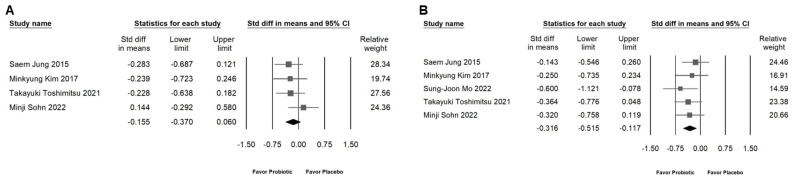
This figure presents a forest plot that delineates the effects of *L. plantarum* supplementation on the L4 abdominal fat area. The plot is organized into two sections: (**A**) demonstrates the *L. plantarum*’s impact on the subcutaneous fat area, and (**B**) shows its influence on the visceral fat area. The horizontal line through the square illustrates the 95% confidence interval, while the diamond symbol summarizes the overall effect size [12,13,19,20,21].

Although *L. plantarum* does not have a significant impact on cholesterol (Figure 5A; SMD: −0.239, 95% CI: −0.512 to 0.034; *I*^2^ = 64.004%, *p* = 0.007), TG (Figure 5B; SMD: −0.244, 95% CI: −0.478 to −0.009; *I*^2^ = 57.859%, *p* = 0.027), HDL (Figure 5C; SMD: −0.106, 95% CI: −0.307 to 0.096; *I*^2^ = 35.350%, *p* = 0.146), LDL (Figure 5D; SMD: −0.215, 95% CI: −0.488 to 0.058; *I*^2^ = 64.164%, *p* = 0.007), or subcutaneous fat areas at the level of L4 (Figure 6A; SMD: −0.155, 95% CI: −0.370 to 0.060; *I*^2^ = 0%, *p* = 0.489), it can be observed to reduce visceral fat areas slightly significantly (Figure 6B; SMD: −0.316, 95% CI: −0.515 to −0.117; *I*^2^ = 0%, *p* = 0.742, *p* = 0.742).

### 2.6. Effects of L. plantarum on Plasma Glucose, Serum Insulin, and Systolic and Diastolic Blood Pressure

**Figure 7:**
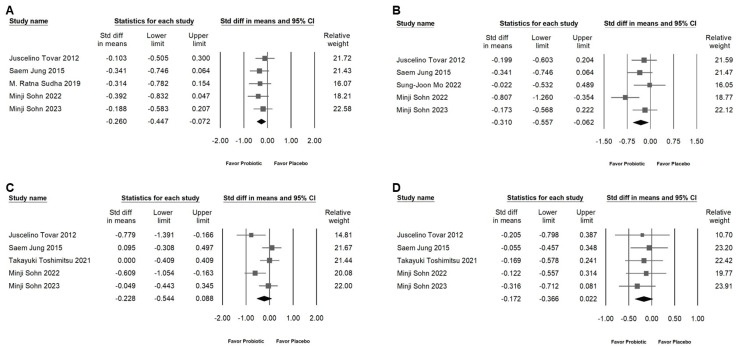
This figure showcases a forest plot illustrating the effects of *L. plantarum* supplementation on various metabolic parameters. The plot is structured into four parts: (**A**) highlights the impact on blood glucose levels, (**B**) examines changes in serum insulin levels, (**C**) assesses alterations in systolic blood pressure, and (**D**) evaluates the effect on diastolic blood pressure. The horizontal line through the square illustrates the 95% confidence interval, while the diamond symbol summarizes the overall effect size [10,11,12,13,18,19,21].

Lactobacillus plantarum has been observed to reduce blood glucose levels (Figure 7A; SMD: −0.260, 95% CI: −0.447 to 0.072; *I*^2^ = 0%, *p* = 0.867) and stabilize insulin expression levels (Figure 7B; SMD: −0.310, 95% CI: −0.557 to 0.062; *I*^2^ = 39.627%, *p* = 0.157) slightly but significantly. These results suggest that L. plantarum may have the ability to regulate carbohydrate metabolism. Furthermore, L. plantarum does not have a significant effect on systolic (Figure 7C; SMD: −0.228, 95% CI: −0.544 to 0.088; *I*^2^ = 60.698%, *p* = 0.038) and diastolic (Figure 7D; SMD: −0.172, 95% CI: −0.388 to 0.022; *I*^2^ = 0%, *p* = 0.925) blood pressure.

### 2.7. Effects of L. plantarum on Inflammatory Markers

**Figure 8:**
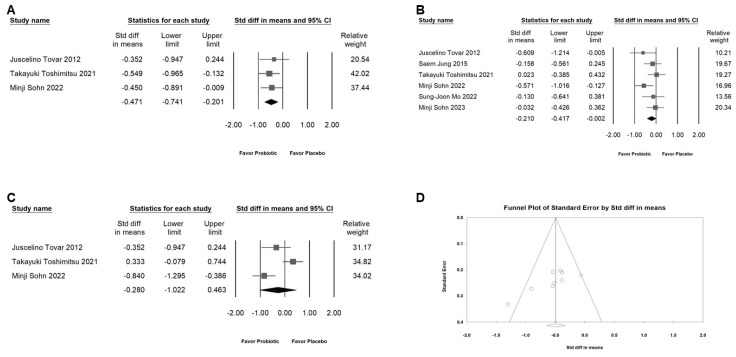
The figure showcases and details the effects of *L. plantarum* supplementation on various inflammatory markers. The horizontal line through the square illustrates the 95% confidence interval, while the diamond symbol summarizes the overall effect size. Designed for straightforward interpretation, the plot is divided into four distinct parts: (**A**) shows the effects on IL-6 levels, (**B**) reports on changes in hs-CRP levels, (**C**) outlines alterations in TNF-α levels, and (**D**) includes a funnel plot summarizing the findings from all studies included. In Figure (**D**), the lines typically represent the confidence intervals around the effect estimates, illustrating the range within which the true effect size is expected to lie. The circles represent individual studies included in the meta-analysis, with their size potentially corresponding to the weight or sample size of the study; larger circles indicate studies with more weight or larger sample sizes. The diamond symbol represents the overall effect estimate derived from the meta-analysis. The center of the diamond indicates the pooled effect size, while the width of the diamond reflects the confidence interval for this estimate [11,12,13,18,19,21].

The impact of *L. plantarum* on inflammatory markers is depicted in Figure 8A–C. *L. plantarum* moderately downregulated the expression level of IL-6 (A; SMD: −0.471, 95% CI: −0.741 to −0.201; *I*^2^ = 0%, *p* = 0.862), had a small effect on hs-CRP (B; SMD: −0.210, 95% CI: −0.417 to −0.002; *I*^2^ = 21.881%, *p* = 0.269), but had no significant effect on TNF-α (C; SMD: −0.280, 95% CI: −1.002 to 0.463; *I*^2^ = 85.994%, *p* = 0.001).

### 2.8. Publishing Bias

Egger’s regression analysis revealed the absence of significant publication bias (*p* = 0.01662) in our dataset. The funnel plots, illustrating the SMD for the efficacy of *L. plantarum* in reduced BW, are depicted in Figure 8D.

## 3. Discussion

Our comprehensive meta-analysis, incorporating data from nine randomized controlled trials with a total of 667 participants, confirms our hypothesis that *L. plantarum* supplementation effectively reduces body weight and BMI in healthy adults with obesity. These findings validate *L. plantarum*’s role as a beneficial agent in weight management and underscore its potential to improve metabolic health through the modulation of the gut microbiota. However, variations in study designs, probiotic strains, and intervention durations suggest the need for further high-quality, large-scale randomized controlled trials to fully elucidate the mechanisms underlying the beneficial effects of *L. plantarum* on weight control.

In addition to *L. plantarum*, other *Lactobacillus* strains, such as *Lactobacillus gasseri* (*L. gasseri*) and *Lactobacillus rhamnosus* (*L. rhamnosus*), have also demonstrated anti-obesity effects in various studies. The mechanisms by which these strains exert their effects may differ, with some influencing lipid metabolism and others modulating appetite-regulating hormones. For instance, *L. gasseri* has been shown to reduce abdominal fat and body weight by regulating lipid absorption [22,23], while *L. rhamnosus* can impact body weight by altering gut hormone levels [24]. Our study also found that combining two or more *Lactobacillus* strains has a more pronounced effect on reducing body weight and BMI. These variations highlight the need for further comparative studies to elucidate the specific pathways and efficacy of different *Lactobacillus* strains in obesity management.

According to the World Health Organization, obesity is defined as the abnormal or excessive fat accumulation that may compromise health [25]. BMI is recognized as the most straightforward metric for assessing obesity. Although the primary cause of obesity is the discrepancy between caloric intake and output, differences in gut microbiota between obese and non-obese individuals can affect energy homeostasis [26]. It is increasingly acknowledged that gut microbiota dysbiosis plays a crucial role in the onset of chronic diseases, including obesity [27]. Notably, obese individuals typically have a higher prevalence of *Firmicutes* and a lower prevalence of *Bacteroidetes* compared to their lean counterparts [28]. In contrast, strains such as *L. gasseri* and *L. plantarum* have been associated with weight reduction or anti-obesity effects, particularly pronounced in individuals who are overweight or obese [4]. Our meta-analysis underscores the vital role of *L. plantarum* in weight regulation, aligning with previous research to demonstrate its pronounced effectiveness in diminishing BW and BMI among obese adults.

Recent meta-analyses have shown that the consumption of *L. reuteri* and *L. plantarum* can effectively reduce total cholesterol and LDL levels, though not impacting the TG and HDL level [29]. Our own meta-analysis reveals that *L. plantarum* does not influence cholesterol, TG, LDL, and HDL levels, or the area of abdominal subcutaneous fat, but significantly impacts the area of abdominal visceral fat. Within our analyzed studies, Takayuki and colleagues discovered that *L. plantarum* OLL2712 had negligible effects on obesity-related indicators, except for IL-6 [21]. Conversely, Rahayu and co-authors observed that an indigenous probiotic, *L. plantarum* Dad-13 powder, significantly reduced body weight and BMI in overweight adults [6]. Hence, the impact of *L. plantarum* on metabolic alterations varies across different investigations, indicating that outcomes may hinge on the specific subjects and strains utilized.

Clamp and colleagues reported that the prevention of bacterial-derived substances (such as toxins, proteins, and lipopolysaccharides) crossing the epithelial barrier by probiotics reduces inflammation and enhances insulin sensitivity in the hypothalamus. This, in turn, leads to reduced food intake, subsequently lowering BMI and body weight [30]. Similarly, our study identified that fasting blood sugar levels were significantly reduced at the baseline compared to the placebo, albeit the effect size was small. This reduction could be attributed to the absence of dietary restrictions and physical activity, or the trial period not being long enough to fully ascertain the outcome. Furthermore, a recent meta-analysis indicated that probiotics had a more pronounced effect in lowering blood sugar levels in individuals with diabetes, with only a tendency for a blood sugar lowering effect in non-diabetic individuals [31]. These findings suggest that the expression of inflammatory substances is associated with obesity. A recent study has shown that IL-6 is abundantly secreted by adipose tissue and is regarded as an indicator of chronic inflammation within adipose tissue [32]. CRP, another inflammatory mediator, is produced by both adipocytes and the liver in response to IL-6; circulating levels of hs-CRP are considered markers for cardiovascular disease risk and have been linked to insulin resistance [32]. Furthermore, hs-CRP is positively correlated with obesity, while weight reduction notably decreases circulating hs-CRP levels [33] and is associated with improvements in insulin resistance [34]. Consistent with those studies, our meta-analysis also revealed that *L. plantarum* significantly reduces the expression levels of IL-6 and hs-CRP. This finding is in line with our earlier analysis, suggesting that the intake of *L. plantarum* can lower blood glucose levels and stabilize insulin concentrations in the serum. Of the nine studies in this meta-analysis, eight were conducted in Asian countries (Korea, Indonesia, Japan), and one in Europe (Sweden), with five of these studies being from Korea. Previous research has indicated that gut microbiota composition can be influenced by diet, genetics, and environmental factors, which vary across populations. For instance, the typical Korean diet, which includes fermented foods like kimchi, may enhance the efficacy of probiotic interventions compared to diets less rich in fermented foods [35,36]. Consequently, we further analyzed the impact of *L. plantarum* between Korean and non-Korean individuals (Figure 4) and found no significant difference in the weight-reducing effects between the two groups. Additionally, the Swedish study showed superior results in reducing body weight, LDL, cholesterol, and systolic blood pressure compared to the average results from the Korean studies, despite its small sample size of 44 participants. These differences may be due to variations in dietary habits, genetic backgrounds, and environmental factors. The high intake of fermented foods in the Korean diet could enhance the effects of *L. plantarum*. Conversely, the smaller sample size in the Swedish study might contribute to greater variability in results. Further studies are needed to investigate how ethno-geographic factors influence the effectiveness of probiotics to better understand their global applicability in obesity management.

This meta-analysis brings important findings to light but presents several limitations. Firstly, the limited number and scale of the trials included in our study call for the cautious interpretation of the results. Additionally, our analysis was unable to further assess the efficacy of *L. plantarum* on body weight in female individuals. This is particularly relevant due to females’ underlying hormonal sensitivities, such as those associated with menstruation and menopause, which may merit deeper exploration. Secondly, the demographic composition of participants in the analyzed randomized controlled trials (RCTs) predominantly featured Asian populations, with eight out of nine RCTs conducted in this group. Given the substantial diversity and temporal variability in human gut microbiota, influenced by factors such as age, genetics, and environmental conditions [37], the applicability of our findings across different ethnicities may be constrained, thereby limiting the extrapolation of our results to a more varied global demographic. Thirdly, the potential variability in gut microbiota composition among participants can be influenced by factors such as age, genetics, and environment. Additionally, many of the reported RCTs included older individuals (aged >65), who may have had pre-existing conditions such as hypertension and hyperlipidemia. These participants often received antihypertensive drugs and statins, which could alter inflammatory markers and values of lipids and blood glucose. Such variability may affect the generalizability of our findings and the observed effects of *Lactobacillus plantarum*. Future studies should control for these variables to better isolate the effects of probiotic supplementation. Fourthly, although the reviewed RCTs exclusively investigated the *L. plantarum* strain, discrepancies in probiotic dosages and the composition of placebo substances across the studies could potentially influence the outcomes of the experiments. For example, one RCT included in our analysis [21] employed a placebo yogurt containing various probiotic strains, which might have compromised the observed effectiveness of *L. plantarum*. This is evidenced by multiple instances where the placebo group outperformed the intervention group. Another study within our review reported that a multi-strain probiotic formulation (UB0316) resulted in a greater reduction in BMI and body weight compared to a placebo, suggesting the efficacy of a multi-species probiotic formula in the management of overweight and obesity [10]. Nonetheless, our meta-analysis did not find a significant difference in efficacy regarding BMI and body weight reduction between single- and multi-strain probiotic formulations. This variance in probiotic dosages and placebo compositions introduces additional complexity to the assessment of *L. plantarum*’s effectiveness.

## 4. Methods and Materials

### 4.1. Data Sources and Selection Criteria

Our study involved a systematic search for RCTs evaluating the effects of *L. Plantarum* strains on overweight individuals. We conducted a thorough search across multiple databases, including PubMed, Scopus, Cochrane Library, and Web of Science, covering the literature up to May 2024. The search terms used included “*Lactobacillus Plantarum*”, “overweight”, “adiposity”, “obesity”, and “obese”, with a focus on clinical trials that involved human participants. Our methodology adhered to the Preferred Reporting Items for Systematic Reviews and Meta-Analyses (PRISMA) guidelines Supplementary Materials [38]. We meticulously reviewed the identified articles, including their bibliographies, to uncover additional relevant studies. The inclusion criteria were restricted to studies published in English and excluded case reports, technical reports, conference papers, review articles, letters, editorials, and laboratory-based studies. This meta-analysis was registered at PROSPERO as CRD42024531611.

### 4.2. Selection of Studies

The screening and selection of studies were independently carried out by two researchers, CPL and JYH, with verification provided by a third researcher, RYT. For a thorough analysis, hard copies of all relevant articles were obtained and rigorously examined. The specifics of the study selection process are depicted in the PRISMA flow diagram (Figure 1).

### 4.3. Data Extraction

Data extraction was independently performed by authors CPL and JYH, utilizing a standardized template consistent with the protocols specified in the *Cochrane Handbook*, particularly guideline [39]. The extracted data included essential information such as the names of the study authors, the year and country of publication, inclusion criteria for study subjects, participant demographics (including count and age), study design, details of the interventions, and the outcomes along with the methodologies employed for their measurement.

### 4.4. Outcomes

The primary outcome analysis in this study focused on body mass index (BMI) and body weight. Secondary outcomes included cholesterol, triglycerides (TG), high-density lipoprotein (HDL), low-density lipoprotein (LDL), subcutaneous and visceral fat areas at the level of the fourth lumbar vertebra (L4), plasma glucose levels, serum insulin levels, systolic and diastolic blood pressure, high-sensitivity C-reactive protein (hs-CRP), interleukin-6 (IL-6), and tumor necrosis factor-alpha (TNF-α).

### 4.5. Quality Assessment

The assessment of potential biases in the included studies was independently performed by researchers CPL and CHK, utilizing the Cochrane Collaboration’s Risk of Bias tool for quality evaluation. Discrepancies in assessments were addressed through discussions with a third reviewer, RYT, to reach consensus. A study was considered to have a high risk of bias if it showed risks in one or more domains.

### 4.6. Statistical Analyses

Data from each included study were analyzed by calculating the Standard Mean Difference (SMD) and 95% confidence intervals (CIs) to evaluate the differences in outcomes between the intervention and control (placebo) groups. These SMDs were subsequently integrated using a random-effects model to accommodate study variability. Comprehensive Meta-Analysis software, version 3, was employed for all statistical analyses. Heterogeneity among studies was assessed using the *I^2^* statistic, where values above 50% indicated significant heterogeneity. Publication bias was examined using funnel plots and Egger’s regression test, with a significance threshold set at *p* < 0.05 for all analyses, except for publication bias, where a threshold of *p* < 0.10 was applied. Additionally, subgroup analyses were carried out to pinpoint sources of heterogeneity, and sensitivity analyses were conducted by systematically removing individual studies to verify the robustness of the overall results.

## 5. Conclusions

In conclusion, our meta-analysis reveals the complex effects of *L. plantarum* on body weight, metabolic parameters, and inflammatory markers. While it demonstrates potential benefits in areas like visceral fat reduction, blood glucose management, and modulation of specific inflammatory markers, the overall health impacts of *L. plantarum* appear to be influenced by factors including probiotic strain combinations, as well as the gender and age of individuals. These insights underscore the need for targeted research to refine probiotic formulations and interventions, potentially increasing their therapeutic efficacy across diverse demographic groups. Future studies should aim to overcome the methodological limitations identified in our analysis to draw more definitive conclusions about the health benefits of *L. plantarum*.

## Figures and Tables

**Table 1:** Characteristics of included studies.

Author (Year)/Country	Inclusion Criteria	Sample Size (% of Male)/Age	Study Design	Placebo Used	Intervention Probiotics/Follow-Up Time	Main Results	Secondary Outcome Measurement
multi-strain							
Tovar (2012) [18]/Sweden	Healthy (BMI of 25–33 kg/m^2^)	44 (18)/ M: 63.6 ± 1.9 F: 63.2 ± 0.8	RCT/ crossover trial	Control diet	1. Antioxidant and phenolics food. 2. Omega-3 fatty acid. 3. *L. plantarum Heal19*, *DSM 15313*. 4. Low-glycemic-impact food/4 weeks	BW reduced to 78.0 ± 1.3 kg (−1.8%; *p* < 0.0001)	Cholesterol (mmol/L), TG (mmol/L), Apolipoproteins (g/L), Insulin (mU/L), Glucose (mmol/L), HbA1c (%), Free fatty acids (mmol/L), Blood pressure (mm Hg), hs-CRP (mg/L), and Inflammatory markers
Jung (2015) [19]/ Korea	Non-diabetic (fasting blood glucose <126 mg/dL and 2 h blood glucose <200 mg/dL) and overweight (BMI between 25 and 30 kg/m^2^)	P: 46 (34.8) I: 49 (36.7)/ P: 37.8 ± 1.63 I: 40.1 ± 1.48	RCT/ double-blind/ placebo-controlled	Control powder (did not contain probiotics)	2 g of powder of two probiotic strains, *L. curvatus HY7601* and *L. plantarum KY1032*, each at 2.5 × 10^9^ cfu, twice a day (immediately after breakfast and dinner)/12 weeks	1. BMI reduced to 26.8 ± 0.23 kg/m^2^ (*p* < 0.001). 2. BW reduced to 72.9 ± 1.30 kg (*p* < 0.001)	Cholesterol (mg/dL), TG (mg/dL), Insulin (mU/L), Glucose (mg/dL), Blood pressure (mm Hg), and Hs-CRP (mg/dL)
Kim (2017) [20]/ Korea	Non-diabetic and overweight individuals	P: 34 I: 32/ no mention	RCT/ double-blind/ placebo-controlled	Control powder (did not contain probiotics)	2 g of probiotic powder twice a day containing *L. curvatus HY7601* (2.5 × 10^9^ cfu) and *L. plantarum KY1032* (2.5 × 10^9^ cfu)/12 weeks	1. BMI reduced (0.23 ± 0.11 kg/m^2^; *p* < 0.05). 2. Body weight reduced (0.60 ± 0.30 kg; *p* < 0.05)	Abdominal fat areas, Total fat mass, Abdominal fat area, and Metabolic profiling of plasma
Sudha (2019) [10]/ India	Non-diabetic male or female between 30 and 65 years of age; BMI between 25 and 32 kg/m^2^; female, not currently pregnant or breast feeding	P: 36 (52.7) I: 35 (37.1)/ P: 41.3 I: 43.5	RCT/ double-blind/ placebo-controlled	Excipient maltodextrin	UB0316 (*L. salivarius UBLS22*, *L. casei UBLC-42*, *L. plantarum UBLP-40*, *L. acidophilus UBLA-34*, *B. breve UBBr-01*, and *B. coagulans Unique IS2*, 5 × 10^9^ cfu each, and prebiotic, fructo-oligosaccharide, 100 mg), 2 capsules per day/12 weeks	1. BMI reduced to 27.4 ± 2.10 kg/m^2^ (−2.83%; *p* = 0.0001). 2. BW reduced to 64.5 ± 7.17 kg (−2.40%; *p* < 0.0001)	Cholesterol (mg/dL), TG (mg/dL), HDL (mg/dL), LDL (mg/dL), VLDL (mg/dL), Glucose (mg/dL), Quality of life, and Waist-to-hip ratio
Mo (2022) [13]/ Korea	Non-diabetic male or female between 19 and 65 years of age; BMI between 23 and 35 kg/m^2^; fasting blood glucose <126 mg/dL	P: 29 (72.4) I: 30 (83.3)/ P: 39.34 ± 1.61 I: 35.7 ± 1.44	RCT/ double-blind/ placebo-controlled	350 mg capsule (250 mg lactose, 5.57 mg crystalline cellulose, 3.5 mg SiO_2_, and 7 mg magnesium stearate)	350 mg capsule contained 250 mg *L. curvatus HY7601* and *L. plantarum KY1032* (5 × 10^9^ cfu), 5.57 mg crystalline cellulose, 3.5 mg SiO_2_, and 7 mg magnesium stearate/12 weeks	1. BMI reduced to 26.73 ± 0.51 kg/m^2^ (*p* < 0.001). 2. BW reduced to 78.74 ± 2.21 kg (−2.40%; *p* = 0.001)	Cholesterol (mg/dL), TG (mg/dL), HDL (mg/dL), LDL (mg/dL), Leptin (ng/mL), Insulin (μIU/mL), Waist circumference (cm), Hip circumference (cm), and Body fat mass
single-strain							
Rahayu (2021) [6]/ Indonesia	BMI equal to or greater than 25, no history of gastrointestinal disorder	P: 30 (40) I: 30 (40)/ P: 44.67 ± 5.66 I: 44.07 ± 6.23	RCT/ double-blind/ placebo-controlled	Skimmed milk	1 g of skimmed milk powder containing the probiotic *L. plantarum Dad-13* of 2 × 10^9^ cfu/90 days	1. BMI reduced to 32.57 ± 5.01 kg/m^2^ (*p* = 0.04). 2. BW reduced to 83.14 ± 14.71 kg (−2.40%; *p* = 0.04)	Cholesterol (mg/dL), TG (mg/dL), HDL (mg/dL), LDL (mg/dL), Gut microbiota composition, and Fecal pH
Toshimitsu (2021) [21]/ Japan	Non-diabetic and overweight individuals (BMI of 25–30 kg/m^2^)	P: 46 (71.7) I: 46 (65.2)/ P: 44.7 ± 8.2 I: 45.5 ± 10.7	RCT/ double-blind/ placebo-controlled	112 g of placebo yogurt	Heat-treated *L. plantarum OLL2712* (>5 × 10^9^ cells/112 g of yogurt)/12 weeks	1. BMI of 27.5 ± 1.4 kg/m^2^. 2. BW of 74.6 ± 8.3 kg	Cholesterol (mg/dL), TG (mg/dL), HDL (mg/dL), LDL (mg/dL), Waist circumference (cm), Hip circumference (cm), Waist-to-hip ratio, Blood pressure (mm Hg), HbA1c (%), and Inflammatory markers
Sohn (2022) [12]/ Korea	Healthy men and women aged 20 to 65 years with a BMI of 25–30 kg/m^2^	P: 40 (40) I: 41 (39)/ P: 45.5 ± 10.0 I: 47.8 ± 11.7	RCT/ double-blind/ placebo-controlled	Equivalent placebo	Two daily allocations of 2 × 10^9^ cfu of *L. plantarum K50* (LPK; total of 4 × 10^9^ cfu/day)/12 weeks	1. BMI of 27.0 ± 1.7 kg/m^2^ (*p* = 0.572). 2. BW of 74.2 ± 10.0 kg (*p* = 0.726)	Cholesterol (mg/dL), TG (mg/dL), HDL (mg/dL), LDL (mg/dL), Insulin (mU/L), Glucose (mmol/L), Waist circumference (cm), Blood pressure (mm Hg), hs-CRP (mg/L), and Inflammatory markers
Sohn (2023) [11]/ Korea	Healthy adult with a BMI of 25–30 kg/m^2^	P: 49 (57) I: 50 (58)/ P: 40.1 ± 10.5 I: 40.2 ± 11.2	RCT/ double-blind/ placebo-controlled	Placebo capsules contained 100% of maltodextrin	Once daily ingested *L. plantarum strain LMT1-48* capsules contained 10% of LMT1-48 (1 × 10^10^ cfu) and 90% of maltodextrin/12 weeks	1. BMI of 27.0 ± 1.7 kg/m^2^ (*p* < 0.05). 2. BW of 75.7 ± 9.2 kg (*p* < 0.05)	Cholesterol (mg/dL), TG (mg/dL), HDL (mg/dL), LDL (mg/dL), Waist circumference (cm), Blood pressure (mm Hg), Insulin (mU/L), Glucagon (pg/mL), and hs-CRP (μmol/L)

BMI: body mass index; BW: body weight; F: females; HDL: high-density lipoprotein; hs-CRP: high-sensitivity C-reactive protein; I: intervention; IL-6: interleukin-6; LDL: low-density lipoprotein; M: males; P: placebo; RCT: randomized controlled trial; TG: triglycerides; TNF-α: tumor necrosis factor-alpha.

## Data Availability

All data generated or analyzed during this study are included in this published article.

## Supplementary Materials

The following supporting information can be downloaded at: https://www.mdpi.com/article/10.3390/ijms25147608/s1. Reference [38] are cited in the supplementary materials.

## Abbreviations

BMI, body mass index; CI, confidence interval; HDL, high-density lipoprotein; hs-CRP, high-sensitivity C-reactive protein; IL-6, interleukin-6; L4, level of the fourth lumbar vertebra; LDL, low-density lipoprotein; *L. plantarum*, *Lactobacillus plantarum*; PRISMA, Preferred Reporting Items for Systematic Reviews and Meta-Analyses; PROSPERO, Prospective Register of Systematic Reviews; RCT, randomized clinical trial; SMD, standard mean difference; TG, triglycerides; TNF-α, tumor necrosis factor-alpha.
